# Censoring chemical data to mitigate dual use risk

**DOI:** 10.1039/d5dd00512d

**Published:** 2026-07-06

**Authors:** Quintina Campbell, Jonathan Herington, Andrew D. White

**Affiliations:** a Department of Chemical Engineering, University of Rochester Rochester New York USA; b Department of Health Humanities & Bioethics, University of Rochester Rochester New York USA jonathan.herington@rochester.edu; c Edison Scientific Inc. San Francisco CA USA andrew@edisonscientific.com

## Abstract

Machine learning models have dual use potential, potentially serving both beneficial and malicious purposes. The development of open-source models in chemistry has specifically surfaced dual use concerns around toxicological data and chemical warfare agents. We discuss a chain risk framework identifying three misuse pathways and corresponding mitigation strategies: inference-level, model-level, and data-level. At the data level, we introduce a noising method to increase prediction error in specific desired regions (sensitive regions). Our results show that selective noise induces variance and attenuation bias, whereas simply omitting sensitive data fails to prevent extrapolation. These findings hold for both molecular feature multilayer perceptrons and graph neural networks. Thus, noising molecular structures represents a step toward enabling safer sharing of potential dual use molecular data.

## Abstract 2

Selectively noising chemical datasets reduces the model accuracy in sensitive regions, offering a data-level approach to mitigate dual use risks in predictive chemistry.

## Graphical abstract

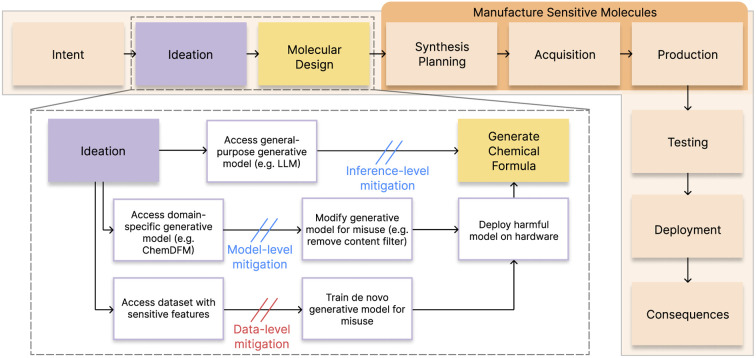

## 1 Introduction

Machine learning in chemistry has become increasingly open and accessible to any individual with a computer and basic knowledge of coding.^1–4^ As with nearly every other novel technology, there are societal risks and opportunities. Easy-to-use ML tools, as beneficial as they can be, have a “dual use” as easy-to-use tools for doing harm. Following the nomenclature used in biology to identify “dual use research of concern”,^5^ we term these harms the dual use risks of predictive chemistry (DURPC).

The “dual use problem” refers to the fact that scientific research “has the potential to be used for harm as well as for good”.^6^ While the vast majority of attention in the last two decades has been on dual use risks generated by research in biology^7–10^ and strong artificial intelligence,^11,12^ chemistry may also pose risks. In chemistry, research investigating toxicity, flammability and some drug properties has caused concern as possible vectors for dual use risks^13,14^ and is the basis of long standing restrictions on the manufacture or possession of certain compounds or precursors.^15,16^

Concern over the dual use risks posed by chemistry are being accelerated by machine learning. Cutting edge large language models (LLMs) can answer and solve graduate level scientific questions,^17^ and can automate even more challenging tasks when they are incorporated in agent frameworks.^18–20^ These advanced capabilities have simultaneously raised safety concerns,^21,22^ including concrete examples where LLMs have assisted in synthesizing dangerous compounds.^19,23,24^ These risks are potentially compounded by open-weight models, which are not subject to active moderation of inputs and outputs. While open-weight LLMs can have safety features integrated into the model itself, others have demonstrated that these safety measures can be extracted or circumvented.^25,26^ Finally, visual language models (VLMs), should not be overlooked in the context of dual use risks, as it is possible that hazardous information, such as a recipe for nerve gas, can be passed in a form of images. Visual modality can bypass the safety constraints of LLMs.^27^ The proliferation of different risks, mitigation strategies, and counter-strategies has made it difficult for institutions and individuals to identify appropriate mechanisms for controlling DURPC.

**Fig. 1:**
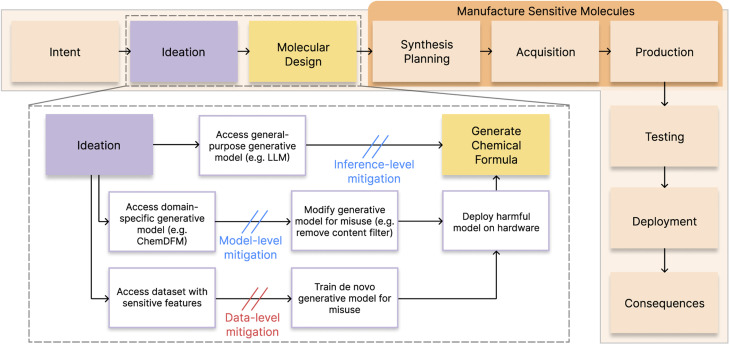
A chain risk framework for dual use risks in predictive chemistry (DURPC). Starting with an actor with intent to do harm (top left), each barrier must be overcome for the consequences to be realized (bottom right). Three distinct pathways are provided for the step between ideation and generating a novel chemical structure. Points of intervention and general mitigation strategies are identified by cross-hatches. Our proposed intervention to reduce DURPC (*i.e.* data-level mitigation) is highlighted in red.

In order to manage DURPC effectively, we develop a more detailed model of the risk landscape for computational chemistry and its ethical dimensions. Prior models for dual use risks have tended to focus on the life sciences^13,28,29^ and we extend a simple chain risk framework, previously used in the context of biological risk.^30^ On this model (see Fig. 1), actors with intentions to cause harm must undertake multiple steps to realize their intent – from settling on a method of causing harm (*i.e.* explosives, toxins, biological agents, *etc.*), through design, manufacture, testing and deployment. Each step poses a barrier to an agent realizing harm, and overcoming each barrier requires a certain level of expertise or access to resources (*i.e.* “agent power” according to Sandberg and Nelson).^30^ This includes how easy it is to source precursors or materials, the level of expertise needed to use the technology maliciously, and the mechanisms for deploying resulting weapons. Steps unique to chemistry tasks are manufacturing steps, where the actor would need to identify precursors to produce the harmful chemical formula by synthesis planning, acquire the necessary precursors and equipment, and synthesize the harmful compound, then purify and scale it up to the desired quantity. By explicitly identifying the barriers that different kinds of agents (*i.e.*, individuals, small organizations, or states) must overcome to produce a chemical weapon, this model helps identify the most effective points for policy interventions to reduce DURPC.

In this paper, we focus on mitigation strategies to prevent the design of novel chemical structures that pose dual use risks. We distinguish between low, moderate and high resource agents in order to help characterize different pathways towards generation of novel chemical structures using generative models. We characterize Low resource agents as ordinary laypeople without a sophisticated understanding of machine learning or chemical synthesis, and without access to resources beyond those available in the consumer retail market. Moderate resource agents are committed, reasonably knowledgeable individuals or small groups with moderate access to resources (*i.e.*, lab equipment or high performance computing resources) that may require knowledge of specialized technical retailers in order to synthesize novel compounds (*i.e.*, chemical synthesis companies). A classic example of such groups is the Aum Shinrikyo cult, which manufactured and released Sarin into the Tokyo subway in 1995.^31^ High-resource agents are so-called “state-level actors”: committed organizations with the scope to recruit subject-matter experts, gather equipment or precursors on an industrial scale, and the time and discipline to engage in multi-year research and development. The risks posed by this last category may be particularly difficult to control, since state-level actors may be able to back-engineer or reproduce their own datasets and models and thus circumvent technical efforts to prevent misuse. Our goal is to limit DURPC for low to moderate resource agents.

A final consideration is the ethical trade-offs involved in any intervention to limit DURPC.^9^ In general, the scientific community wants to make toxicity data generally available, so that researchers and practitioners can generate their own predictors to generate beneficial knowledge about novel molecules. This position aligns with scientific norms of openness and reproducibility, which have historically accelerated innovation and safety improvements in chemistry. Some philosophers^7,32^ of science have argued that traditional scientific norms may require recalibration towards “precautionary” approaches when technologies offer unprecedented potential for harm alongside uncertain benefits. On the other hand, precautionary approaches can be self-defeating if they prevent research which defends against risks other than DURPC which may be more likely or more harmful.^33^ Maintaining the accuracy of toxicity predictors is critical to mitigating unintentional harm from generative models which identify novel, potentially useful chemical compounds. In this respect, the effect of DURPC interventions on the overall accuracy of predictors, or their accuracy with respect to different zones of chemical space, must be carefully evaluated.

## 2 Related works

**Fig. 2:**
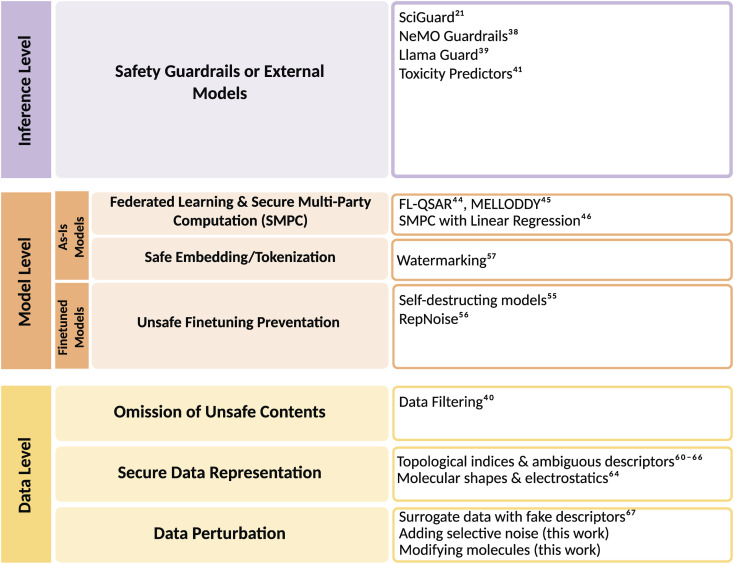
Summary of strategies for mitigating DURPC, categorized into three levels based on where the strategy is implemented: dataset, model, and inference.

Mitigating DURPC shares a similar basic structure as ensuring privacy and safety in machine learning and artificial intelligence (AI): it involves identifying classes of sensitive information and generating strategies to effectively limit the disclosure of such information to end users. In the context of general AI privacy and safety, the classes of sensitive data might include demographic information, socioeconomic status, hate speech, criminal planning, to list a few.^34^ Meanwhile, in chemistry, classes of sensitive information would consist of toxicity, highly energetic materials, nuclear or biological weapon precursors, and psychoactivity (narcotics), often categorized within the “chemical, biological, radiological, nuclear” (CBRN) hazard category,^35,36^ and sometimes with high yield “explosives” (CBRNE).^37^ Strategies to counteract and mitigate dual usage of this information can be broadly categorized into three approaches: inference-level mitigation, model-level mitigation, and data-level mitigation (Fig. 2).

### 2.1 Inference-level mitigation

Inference-level methods to mitigate dual use risks, also known as system-level mitigations, are front line defenses and often consist of external safety guardrails^38–40^ or models.^21,41^ These external techniques would detect and control potential harm in user input, model output, or both. In particular, Llama Guard^39^ can detect inputs or outputs that facilitate the construction of illegal weapons and regulated substances. An external LLM agent SciGuard^21^ is designed to detect and control sensitive information in scientific models. The limitations with the strategies above are that they do not mitigate the risks posed by open-source models, and are prone to be bypassed by medium and high resource agents by jailbreaking or similar.

### 2.2 Model-level mitigation

Model-level mitigations can be divided into two subcategories based on their objectives: (1) model training or architecture designated to secure sensitive data or model weights, and (2) safety alignment of models to lower the probability of unsafe outputs in the first place. To secure data, federated learning is commonly used and allows data owners to collaboratively train a model by aggregating their model weights into a global model without sharing their datasets.^42,43^ FL-QSAR and MELLODDY are few instances of federal learning models in science.^44,45^ Another similar method is to use secure multi-party computation (SMPC/MPC), which has been used for linear regressions with chemical data.^46^ The limitations of federated learning and MPC are that while they may secure chemically sensitive information classes in data, they do not align models away from the sensitive regions in the chemical space.

For model-level alignment of AI, reinforcement learning with human feedback (RLHF) or other forms of reinforcement learning with safety cost functions are commonly used to reduce safety risks.^47–53^ RLHF models become more challenging to red-team as they scale, whereas other model types show flat trends when scaled up.^54^ To prevent harmful fine-tuning, some defense mechanisms are “self-destructing” models to slow the convergence of harmful training^55^ and representation noising, *i.e.* RepNoise,^56^ to reduce and remove harmful representations within models while fine-tuning. Additionally, safe embeddings or tokenization, such as the ‘watermarking’ framework,^57^ can tailor the model away from generating unsafe contents. However, high computational costs are a common limitation when utilizing reinforcement learning or other fine-tuning approaches to aligning models for chemical safety or AI safety in general.

### 2.3 Data-level mitigation

Finally, mitigating DURPC at the data level can be achieved by omission of sensitive data (also called data filtering) or data obfuscation. Data filtering, commonly used in large language models,^40^ removes bias and unsafe content from training data to prevent introducing sensitive information to models. However, this is challenging in regression tasks for chemical data, where models can extrapolate to novel, potentially harmful chemicals, as seen in bioactivity models.^58^

Data obfuscation can be done by using an ambiguous representation, surrogate data, or data perturbation. Research on censoring chemical data dates back to a 2005 ACS symposium, which discussed the exchange of chemical data without revealing molecular structures.^46,59–72^ While initially aimed at protecting intellectual property, this work can also apply to mitigate the safety risks associated with revealing chemical structures. A key finding was that even minimal chemical information can expose structures, leading to reluctance to share pharmaceutical data.^59^ For example, molecular masses can be sufficient to infer structures.^60^ Later studies explored which descriptors could or could not reveal structures.^60^

Some studies propose ambiguous representations with high degeneracy (multiple molecules per representation) to censor chemical structures. Examples include topological indices,^61,62^ substructural fragment matrices,^63^ molecular shape or electrostatics descriptors,^64^ chemical relationships,^65^ and identical descriptors for distinct molecules to cause collision and confusion.^66^ However, these methods do not steer a model away from sensitive classes and thus possibly fail to prevent it from predicting sensitive classes. Furthermore, a study shows that reverse engineering a structure from a single descriptor is possible, suggesting that descriptors may not be reliable methods to censor sensitive information and suggesting surrogate data as an alternative.^67^ These surrogate data replace the original molecule's fingerprints with those of similar molecules to mask chemical structures while maintaining model performance.

### 2.4 Assessing dual use risks

While this work focuses solely on mitigating dual use risks, another equally important aspect of AI safety is assessing dual use risks. Most prior work has focused on identifying and evaluating these risks, even more in LLMs, commonly performed by red teaming^54,73–75^ and benchmarking.^34,76^ A risk category containing CBRN(E) is included in a taxonomy for red teaming^35^ and benchmarks.^21,36,37,77^ Compared to risk assessment, there is only a sparse literature focused on developing techniques to mitigate DURPC in machine learning and artificial intelligence. Nonetheless, many frameworks for AI safety propose multifaceted approaches rather than implementing a single technique,^22,78^ and the same is likely to be true for mitigating DURPC in machine learning and artificial intelligence.

### 2.5 Our work: adding selective noise to the chemical data

Machine learning models are sensitive to the distribution, imbalance and noise of the dataset. As a method for mitigating DURPC, we propose selective addition of noise to specific portions of chemical datasets. This data-level mitigation approach adds noise to the labels or features of only those data classes that are flagged as sensitive, such as highly flammable chemicals, toxins, or nerve agents. This method of selective noising of data points in the dataset can effectively contribute to mitigating dual usage for two main reasons.

Firstly, we apply selective noise in datasets as a step toward enabling data sharing. As demonstrated by the bioactivity model,^58^ removing sensitive data points does not prevent the model from making sensitive predictions. Data collection is costly in terms of time and resources, and data scarcity is a major challenge in building machine learning models for science.^79^ This has led to growing interest in low-shot, zero-shot, and other small-data learning approaches.^80,81^ When data can be shared safely, researchers gain access to valuable datasets while minimizing DURPC, thus fostering open collaboration and the advancement of chemical and biological technologies.

Secondly, this method provides a model-agnostic approach to mitigating the risks of DURPC across the architectures evaluated here, though its generalizability to other architectures, including equivariant architectures, message-passing networks, and molecular transformers remains to be established. This property is important given the increasing accessibility of machine learning tools and resources; individuals can build their own models and use LLMs to generate code to train them.^18^ Inference-level and model-level strategies may limit misuse of existing models, but are insufficient to address potential dual use risks from custom models developed by individuals with access to sensitive data. Data-level mitigation addresses dual use concerns regardless of which architecture is being used.

## 3 Theory

We can write a data generating function (*e.g.* measuring a chemical property) as: f(*x⃑*) = *y*, where *x⃑* is a featurization of the molecule and *y* is the measured property ††For simplicity we treat labels as *y* = *f*(*x⃑*), ignoring the inherent noise term *ε*, in our theoretical analysis, though we include it in all empirical studies to reflect realistic experimental conditions..

In the usual machine learning setting, we receive data 
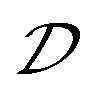
 that are pairs of (*x⃑*_*i*_,*y*_*i*_) from f(*x⃑*) to fit an approximation: *f̂*(*x⃑*; *θ*). That approximation is defined by parameters *θ*, which could be, for example, neural network weights or best-fit linear coefficients.

We can denote the sensitive regions *via* some function *s*(*y*) = 1 or 0, such as *y* > *y*_*t*_, where *y*_*t*_ denotes some threshold for label values to be sensitive. For simplicity, we consider only two classes of data: sensitive and non-sensitive, with *y*_*t*_ representing a constant threshold used to distinguish between the two. The generalization error (expected mean squared error in the test data) is written as *E*_*x,θ*_[(*f̂* − *y*(*x⃑*))^2^], where the expectation is taken over both the test point (*x⃑*) and the parameters (*θ*), which depend on 
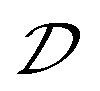
.

Our goal is that:

1 *E*_*x⃑*,*θ*|s = 1_[(*f̂*(*x⃑*; *θ*) − *y*)^2^] ≫ *E*_*x⃑*,*θ*|s = 0_[(*f̂*(*x⃑*; θ) − *y*)^2^]

where *s* = 0 means *s*(*y*) = 0. A baseline approach is to omit the points where *s*(*y*) = 1. For example, when predicting toxicity, the most toxic molecules considered sensitive would be removed from the training data. A clear drawback is that there is no control over the generalization error; we cannot prevent a model from learning *via* extrapolation.

Here we propose to generate noisy data, *i.e.* perturbed data, 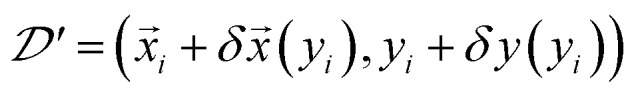, where *δx⃑*(*y*_*i*_) and δ*y*(*y*_*i*_) are controllable probability distributions and are dependent on whether the label *y*_*i*_ resides in the sensitive region. These distributions should be chosen to minimize generalization error in our non-sensitive region and maximize in our sensitive region. Among the noise types previously evaluated, zero-mean Gaussian noise is reported to be particularly effective in reducing deep learning model performance.^82^

A direct analysis of eqn (1) depends on the training procedure: 
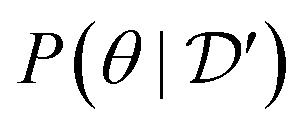
.^83^ Some intuition can be found by assuming that θ could be separated into *s* = 1 and *s* = 0 regions and then using the bias-variance decomposition of the generalization error:^84^

2 *E*_*θ*_[(*f̂*(*x⃑*; *θ*) − *y*))^2^] = (*E*_*θ*_[*f̂*(*x⃑*; *θ*)] − *y*)^2^ + *V*_*θ*_[*f̂*(*x⃑*;*θ*)]

where *V*_*θ*_ is the variance component and we have used our assumption that *y*, a test point label, does not have noise added. Based on eqn (2), a hypothesis is drawn where supposedly 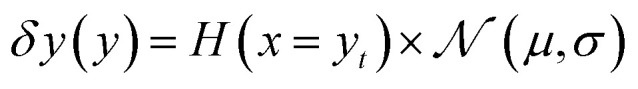 is a zero-mean Gaussian distribution,^82^ then *θ* in the sensitive region would tend towards zero bias but high variance (as set by δ*y*(*y*)). If *δx⃑*(*y*) is a zero-mean Gaussian distribution, the bias would not tend towards zero due to regression dilution/attenuation.^85^ Instead, the bias decreases to flattening *f̂*(*x⃑*; *θ*) = 0. The variance also increases with *δx⃑*(*y*).

Based on this analysis, we expect that adding noise in *x* will increase model bias and variance in the sensitive region. Adding noise to *y* alone should not affect bias, but increase variance. This analysis is based on the assumption about the separability of *θ* into *s* = 1/*s* = 0 regions and may not hold. Hence, our results below explore this empirically in increasingly complex models.

## 4 Methods

We apply and assess selective noise injection to data points that are deemed ‘sensitive’ by adding zero-mean Gaussian noise to numerical features and labels. First, we define a sensitivity threshold *y*_*t*_, which partitions the dataset into sensitive (*s*(*y*_*i*_) = 1) and non-sensitive (*s*(*y*_*i*_) = 0) data. Initially, this threshold is fixed to zero. In later experiments, *y*_*t*_ is dynamically set based on the sensitivity split parameter *α*. The value of *α* determines the fraction of data considered sensitive. Specifically, *y*_*t*_ is the label value that separates the top-α fraction of the dataset. For example, if *α* = 0.1, the top 10% of data points with the highest labels are classified as sensitive and *y*_*t*_ is the label value that separates them from the rest.

Once *y*_*t*_ is determined, label noise *δy*(*y*) or feature noise *δx⃑*(*y*) is selectively applied to sensitive data in both training and validation sets. The standard deviations of zero-mean Gaussian noise, referred to as “noise level,” control the extent of selective perturbation. We evaluate the model accuracy on unseen raw sensitive data points, as well as on non-sensitive data, to assess how well the model generalizes across both regions.

To add feature noise to selected SMILES representations, we replace the molecules with structurally similar ones using the “superfast traversal, optimization, novelty, exploration, and discovery” (STONED) method^86^ with local chemical space generation as described by Wellawatte *et al.*^87^ The replacement molecules serve as chemically-informed feature noise, not merely as neutral mathematical noise carriers but rather as chemistry-aware perturbations to confound machine learning models in the chemical space. The similarity of the replacement molecule to the original molecule is measured by the Tanimoto similarity and ultimately determined by the noise level. All SMILES representations are then converted to molecular graphs for GCN training. Further details on SMILES feature noise can be found in SI. The model setup and training specifications can also be found in SI.

## 5 Results

To evaluate the effectiveness of selective noise in censoring sensitive data, we assess the model performance on three increasingly complex tasks: (1) polynomial regression on 1D synthetic data, (2) a multilayer perceptron (MLP) on high-dimensional synthetic data, and (3) a graph convolutional network (GCN)^88^ on an experimental molecular dataset to predict lipophilicity. For each task, we assess the performance numerically using eqn (1) and visually with a parity plot (*y vs. f̂*(*x*)) on unseen raw data.

### 5.1 Visualizing selective noise with 1D polynomial regression

**Fig. 3:**
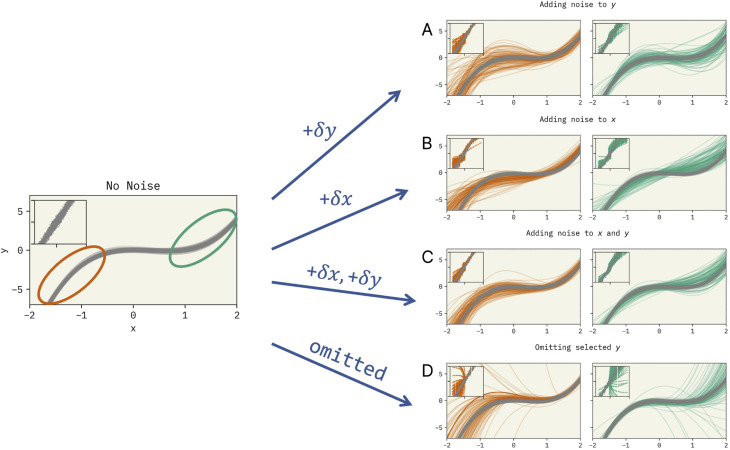
Fitted cubic curves after applying four different training data perturbations for 100 trials. Left: the gray curve represents ground truth model (*x*, *y*). Right: models trained on perturbed data. Four perturbation types are applied in the following order: label noise (*δy*(*y*)) (A), feature noise (*δx*(*y*)) (B), and combined label & feature noise (δ*y*(*y*),δ*x*(*y*)) (C), and selective omission (D). Selective perturbation is applied to either of two data regions: data points with negative labels *y* < 0 (orange) and data points with positive labels *y* > 0 (green). Inset figure in each subplot shows a corresponding parity plot on raw unseen data.

The nature of polynomial regression with 1D feature input allows us to visualize the fitted model on the (*x*_*i*_, *y*_*i*_) plot and clearly interpret the differences in the fitting parameters after adding selective noise. Using a synthetic dataset, we applied polynomial regression with least squares to analyze fitted models under three conditions: raw data, noisy data (with three variations of selective noise), and filtered data after omission. Data perturbations, either selective noise or omission, are applied to two different regions of data: negatively labeled data (fitted curves in orange) and positively labeled data (green), as shown in Fig. 3. For reference, curves *f̂*(*x*) fitted to true data 
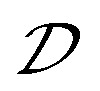
 are shown in gray.

Original data 
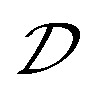
 was generated from a cubic equation *y* = *x*^3^ − *x*^2^ + ε, where 
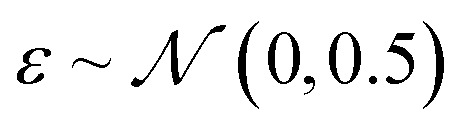
 mimics the small inherent noise. Sensitivity was defined as *s*(*y*) = 1 for data points where *y* > 0 or *y* < 0, as we studied both cases separately. Modified data 
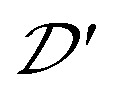
 was generated by adding Gaussian noise: 
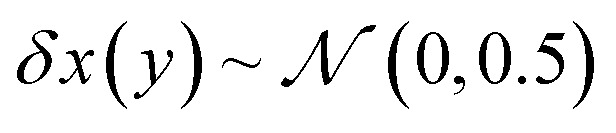
, 
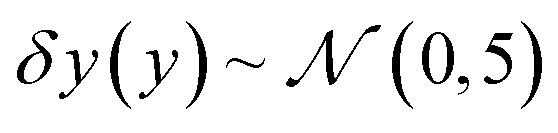
, or their combination *δx*(*y*) + *δy*(*y*) in *s*(*y*) = 1. For combined noise, standard deviations were halved. A cubic fit *f̂*(*x*) was applied to 
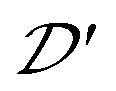
 across 100 trials. Noise levels were calibrated empirically to produce comparable effects on model performance. Feature noise *δx* had a greater impact than the label noise *δy* for the same Gaussian noise level, requiring a higher standard deviation for *δy* to achieve similar levels of performance deterioration. The difference in noise level scaling is due to their fundamentally different mechanisms in inducing bias and variance and are discussed below. In other words, the model is very sensitive to feature noise compared to label noise. In the baseline omission method, removed data points were not replaced, leading to fewer data points for analysis and reflecting the nature of filtered data.

As seen in both fitted curves and parity plots in Fig. 3A, adding selective label noise *δy* to training data increases variance, particularly in the sensitive region. Selective feature noise *δx* induces high bias in the sensitive region, evident from the suppression of curvature in fitted curves and the regional shifts in parity plots. Feature noise also introduces some variance, predominantly in the sensitive region. Selective noise thus allows controlled fitting to specific *y* regions in polynomial regression, aligning with theoretical expectations in Section 3. Applying both label and feature noise leads to increased variance and bias in the *s* = 1 region. The presence of attenuation error in the parity plots (inset figures in Fig. 3B and C) demonstrates that selective noise can specifically control bias at extreme values.

Omitting data points where *y* falls in the sensitive region creates a strong differential in generalization error. In one-dimensional polynomial regression on (*x*_*i*_, *y*_*i*_), the model does not extrapolate beyond the training range, enabling the omission to censor sensitive *y* values. However, we anticipate that deep learning models, such as MLPs, will be able to extrapolate well in high-dimensional settings, making omission less effective.

### 5.2 Selective noise in multilayer perceptrons (MLP) with synthetic data

**Fig. 4:**
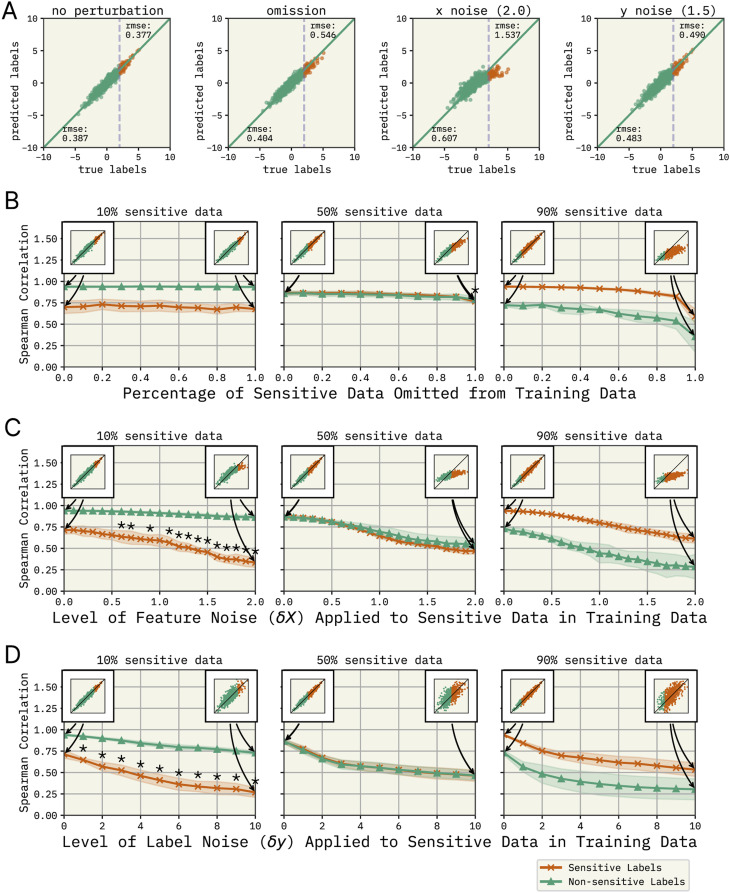
Evaluation of MLP Performance with Selective Noise. (A) A single instance of MLP parity plot after training on each type of data perturbation. We use *y* > 2 here as the threshold for sensitive labels. Noise levels are *n*_*x*_ = 2.0 for features and *n*_*y*_ = 1.5 for labels. (B) Spearman correlation of *y vs. f̂*(*x*) on unseen raw data after training on selectively omitted data, dependent on the amount of sensitive data in the dataset. (C) Spearman correlation on unseen raw data after training on selective feature noise. (D) Spearman correlation on unseen raw data after training on selective label noise. *Note: asterisks indicate noise levels where sensitive region degradation was significantly greater than non-sensitive region (Wilcoxon signed-rank test, *p* < 0.05). Label noise and feature noise degraded sensitive data performance significantly more than omission across nearly all tested noise levels (*p* < 0.05, Wilcoxon signed-rank test), except feature noise at the highest noise level with 90% sensitive data.

We used a multilayer perceptron (MLP) to evaluate a multidimensional case. To generate high-dimensional synthetic data, we initialized an MLP with random parameters and used it to create raw data 
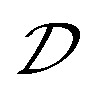
 consisting of (*x⃑*_*i*_,*y*_*i*_) with feature dimension of 50. To maximize error in the sensitive region *s*(*y*) = 1 while minimizing error in the non-sensitive region *s*(*y*) = 0, we tested various levels of selective noise by adjusting the standard deviation of zero-mean Gaussian noise. For omission, we controlled the perturbation effect by varying the percentage of training data omitted in the sensitive region. In addition to using a fixed threshold *y* > *y*_*t*_ (shown in Fig. 4A), we also analyzed different sensitive/non-sensitive splits. Fig. 4B–D presents the results for the 10%, 50%, and 90% splits, showing the effect of noise level adjustments. Parity plots are included as insets to visualize the distribution of both sensitive and non-sensitive labels and to capture changes in distribution due to selective noise.

The baseline omission method has minimal impact on model accuracy, measured by the Spearman correlation of ground truth *y vs.* prediction *f̂*(*x⃑*). Varying the omission percentage provides little control over accuracy for both sensitive and non-sensitive labels. Omission is only effective when a large portion of the dataset is sensitive and at least 80% of these sensitive instances are removed from training and validation data. However, in such case (*e.g.*, a 90% sensitivity split), model accuracy drops significantly – by about 35% lower for sensitive labels and 50% lower for non-sensitive label – due to the small size of the remaining dataset.

Selective feature noise here is defined as 
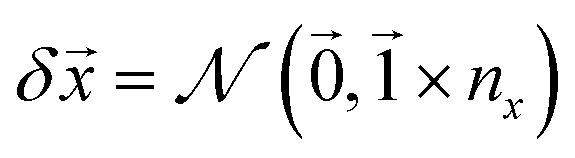
 , where *n*_*x*_ denotes the noise level. Fig. 4C shows that as feature noise increases, the trade-off between selective noise effectiveness (test error for sensitive labels) and desired accuracy (test error for non-sensitive labels) diminishes. At high feature noise (*n*_*x*_ = 2.0) with a 10% sensitivity split, generalization error for non-sensitive labels remains nearly unchanged, while the Spearman correlation for sensitive labels drops from 0.71 to 0.33. With a 50% sensitivity split, accuracy declines by 45% for sensitive labels and 37% for non-sensitive labels. At extreme sensitivity levels (90%), the trade-off becomes more severe: a 30% accuracy drop for sensitive labels *vs.* 60% for non-sensitive labels. This effect is only observed in highly sensitive datasets. Inset parity plots in Fig. 4C reveal attenuation bias, with flattening within the sensitive region. Overall, feature noise provides greater control over accuracy across all sensitivity splits compared to omission.

Label noise is defined as 
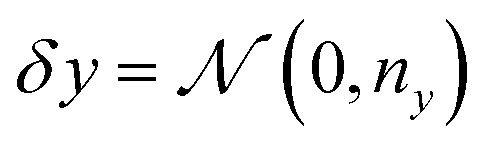
 , where *n*_*y*_ is the noise level. Injecting selective label noise induces a trade-off between model accuracy in both regions, decreasing at roughly the same rate as label noise increases across nearly all sensitivity splits. Inset parity plots in Fig. 4D show that label noise introduces variance across both regions, despite being selectively applied only to sensitive labels in training data.

In summary, for MLP on high-dimensional data, omission has little effect on accuracy except in extremely sensitive datasets, label noise primarily induces variance across both regions, and feature noise causes desired regional attenuation bias in low-to-moderate sensitivity settings.

### 5.3 Selective noise in graph convolutional network (GCN) with experimental data

**Fig. 5:**
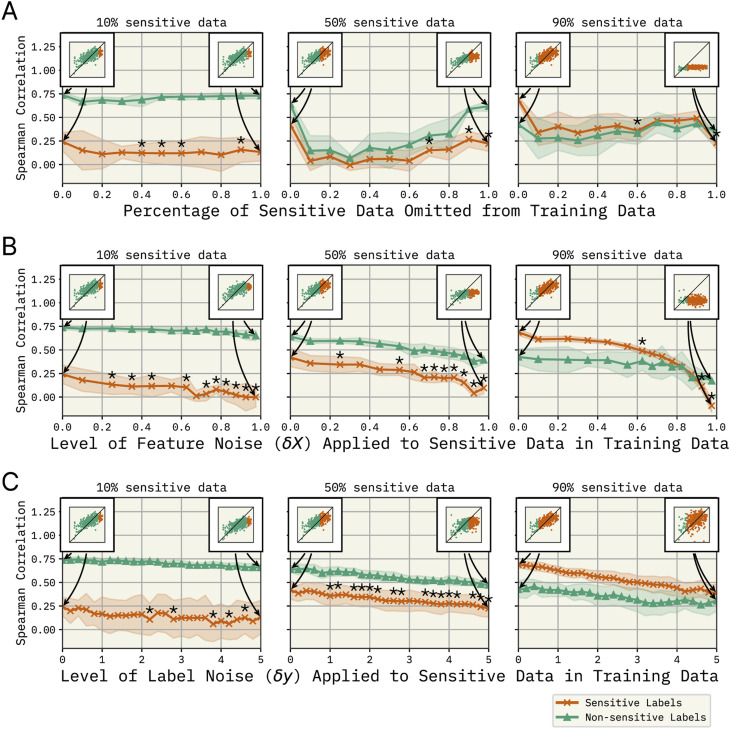
Evaluation of GCN Performance with selective noise. (A) Spearman correlation of *y vs. f̂*(*x*) on unseen raw data after training on selectively omitted data, dependent on the amount of sensitive data in the dataset. (B) Spearman correlation on unseen raw data after training on selective feature noise. (C) Spearman correlation on unseen raw data after training on selective label noise. *Note: asterisks indicate noise levels where sensitive region degradation was significantly greater than non-sensitive region (Wilcoxon signed-rank test, *p* < 0.05). Feature noise caused significantly greater degradation of sensitive data performance than omission at the maximum noise level across all sensitive data proportions, and than label noise for 50% and 90% sensitive data (*p* < 0.05, Wilcoxon signed-rank test).

As the final task, we evaluated the censoring of sensitive data using a graph convolutional network (GCN) trained with selective noise.^88^ We used experimental lipophilicity data, which measures a molecule's preference for octanol over water.^89^ Lipophilicity was selected as a well-established regression dataset in cheminformatics; its drug-like molecules with sufficient molecular size range make it particularly suitable for demonstrating the feature noise method. Unlike synthetic datasets with controlled artificial noise in previous tasks, this experimental dataset contains inherent noise from experimental measurements. The dataset 
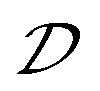
 consists of molecular graphs (*x*) and measured lipophilicity log *D* (*y*). Feature noise cannot be applied as Gaussian noise due to the molecular graph structure. Instead, we replaced molecules with structurally similar ones, where noise level is inversely related to Tanimoto similarity, with higher noise producing more dissimilar molecules (see SI). As in previous models, Gaussian noise was selectively added to labels. We used Spearman correlations to mitigate magnitude effects from the dataset's uneven distribution and sparsity as observed in zero-noise parity plots of our results. However, this does not fully eliminate the influence of the underlying data distribution, resulting in varying initial correlation values across sensitivity splits in Fig. 5.

Omitting sensitive data does not reduce the accuracy of sensitive predictions in the sensitivity splits 10% and 50%, confirming that omission is ineffective for censoring in deep learning models, which extrapolate relatively well for regression tasks. At 50% and 90% sensitivity, omission results vary widely as the impact of randomness in selecting data points outweighs the effect of the omission fraction, making the trends between omission and accuracy difficult to discern.

Compared to omission, selective feature noise effectively reduces accuracy for sensitive predictions across all sensitivity splits (Fig. 5B). The accuracy drop is more pronounced for sensitive labels than for non-sensitive ones: at a 10% sensitivity split, accuracy decreases by 100% for sensitive labels but only 12% for non-sensitive labels, if the noise level is maximized. At the 50% split, the drop is 77% *vs.* 38%. At the 90% split, it is 113% *vs.* 60%, with sensitive label correlation falling slightly below zero (−0.087), hovering near zero as desired for privacy. Feature noise achieves statistically significant selective censoring (marked by asterisks per noise level tested in Fig. 5), a property that is not consistently observed in label noise or omission, which degrades both regions indiscriminately. This selectivity is most pronounced with 10% and 50% sensitive data. At 90% sensitivity split, no method achieves consistent and reliable selective censoring.

Similar to the findings with MLP, label noise induces variance across both regions (Fig. 5C). Its impact is minimal at a 10% sensitivity split but increases at the 90% split, where correlation for sensitive labels steadily declines, indicating some control over predictions.

With GCN, feature noise selectively reduces accuracy for sensitive data while having a smaller effect on non-sensitive predictions, most reliably at low to moderate sensitivity ratios. However, at a high sensitivity ratio, the loss of data utility in the non-sensitive region is substantial (60%). This reflects the difficulty of maintaining selectivity when the sensitive region dominates the dataset. Label noise induces variance rather than attenuation bias but can still impact extreme labels. These effects persist even in experimental datasets with measurement noise. The omission method does not show a clear trend with increasing sensitive data fraction, making its behavior difficult to predict in practice.

**Fig. 6:**
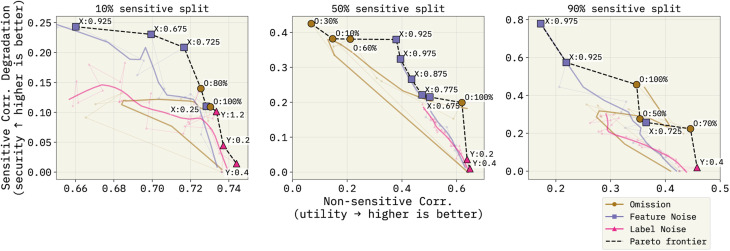
Security-utility Pareto frontier for GCN models. Each curve represents the tradeoff between non-sensitive prediction accuracy (utility) and degradation of sensitive prediction accuracy (security) for each perturbation method, smoothed *via* LOWESS for visual clarity. The Pareto frontier, indicated by the dashed line, is computed on the underlying raw data. Frontier points are annotated with perturbation method (O = omission, X = feature noise, Y = label noise) and corresponding noise level.

To clearly observe the security-utility tradeoff, we conduct a Pareto analysis across all sensitivity ratios in Fig. 6. Feature and label noise demonstrate clear, monotonic tradeoff curves, whereas omission shows no consistent trend, particularly at the 90% sensitive fraction. Nevertheless, across all sensitivity splits, omission at 70–100% consistently appears on the Pareto frontier, suggesting it can preserve considerable utility, although at the cost of security. Label noise dominates the high-utility, low-security end of the frontier across all splits. In the optimal region, where both security and utility are reasonably high, the frontier points are split between omission and feature noise, though which prevails at a given point depends on the unpredictable behavior of omission. Notably, at the 10% sensitive fraction, feature noise dominates the high-security end. These results suggest that feature and label noise offer predictable, monotonic tradeoffs, while omission may behave differently depending on the dataset and architecture.

### 5.4 Adversarial fine-tuning of graph convolutional network (GCN) with selective noise

**Fig. 7:**
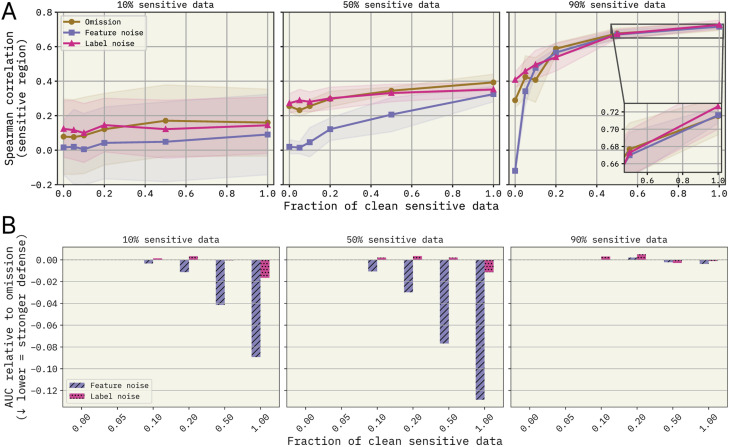
Adversarial fine-tuning of GCN with selective noise. (A) Spearman correlation in the sensitive region is recovered as GCN models are fine-tuned on increasing fractions of clean sensitive data, after being trained with each censoring strategy at maximum intensity (omission: 100%, label noise level: 5.0, feature noise level: 0.975). Shaded bands indicate ±1 standard deviation over 5 trials. (B) Recovery AUC (area under the Spearman correlation *vs.* clean fraction curve) relative to omission, computed as ΔAUC = AUC_noise_ − AUC_omission_ at each fraction of clean sensitive data. A negative value indicates that the censoring strategy has a smaller AUC, implying greater resistance to adversarial fine-tuning than omission.

As a follow-up to the training of GCN models on perturbed datasets, we evaluated which censoring method would be more robust to adversarial fine-tuning given that the adversary had access to partial or the entire sensitive data. In Fig. 7, we fine-tuned the GCN models solely on the increasing fraction of clean sensitive data.

For 10% sensitive data, all censoring methods are relatively effective in preserving model accuracy for sensitive labels, though feature noise produces the lowest correlation across all clean fractions. For 50% sensitive data, feature noise is considerably more resistant to adversarial fine-tuning when the adversary has access to 50% or less of the clean sensitive data, compared to label noise and omission. With 90% sensitive data, all three censoring methods converged rapidly with a small fraction of clean sensitive data, approximately 10%, showing little to no differences among censoring methods.

When the model is originally trained on predominantly sensitive data, it is more prone to adversarial fine-tuning regardless of the censoring type. For training sets with 50% or less sensitive data, feature noise is shown to be the most resistant to such attacks, consistently outperforming label noise and omission. At the 90% sensitive data fraction, despite similar final correlation values among censoring methods, fine-tuning under feature noise required considerably more epochs, with a hard minimum of 85 epochs (mean: 259 ± 94, range: 85–446), compared to label noise (mean: 126 ± 71, range: 12–281) and omission (mean: 160 ± 89, range: 13–396). This suggests that the induced attenuation bias potentially degrades the model in a more fundamental way that requires more clean sensitive data to undo. These results suggest that for datasets with low to moderate sensitive data proportions, a moderate-resource adversary might require a substantial fraction of clean sensitive labels to meaningfully recover predictive accuracy, particularly under feature noise censoring. At high sensitive data proportions, accuracy recovers rapidly regardless of censoring method, though feature noise imposes a higher computational burden on the adversary.

## 6 Discussion

Selective feature noise is demonstrated to be a more effective data-level censoring strategy than label noise and omission, most consistently at lower sensitive data proportions. It induces targeted attenuation bias while remaining the most resistant to adversarial fine-tuning. Crucially, our study shows that simply omitting sensitive data does not prevent extrapolation in deep learning models such as MLP and GCN. However, feature noise also degrades non-sensitive performance, more substantially when applied to highly sensitive datasets (*i.e.* 90% sensitivity ratio), creating substantial tradeoffs between security and data utility, requiring careful deployment considerations.

There are some theoretical and practical limitations to this work. Theoretically, the parameter separability assumption does not hold in practice due to parameter sharing in neural networks, causing noise effects to propagate across regions. This likely explains the observed utility tradeoffs. Also, our approach assumes that sensitive and non-sensitive molecules occupy distinct regions of chemical space, which may not hold for all property types and would require case-by-case validation for practical applications.

Practically, binary sensitivity definitions oversimplify multidimensional real-world risk categories, potentially compounding utility losses through overlapping censor regions. Determining sensitivity thresholds requires an adaptive governance framework, grounded in established international standards such as the Chemical Weapons Convention schedules^16,90^ as a recognized baseline. This categorization should not be a fixed decision, but rather updated through a continuous cycle of multi-stakeholder deliberation among domain experts, security specialists, and the public, given that technological progress consistently outpaces regulatory guidelines.^91,92^ When expert disagreement arises, as empirical evidence suggests is common, structured mechanisms such as multi-member review panels, choosing conservative thresholds, and following default rules for well-characterized compound classes can provide a path forward.^93^

As another practical limitation, effective censoring severely impacts legitimate applications for moderately or highly sensitive datasets (33–60% accuracy drops for non-sensitive predictions), although lower noise levels can partially mitigate this trade-off. Moreover, the molecular feature noise strategy faces a molecular size constraint at the lowest noise level, leading to omission of up to 10% for the lipophilicity dataset due to insufficient highly similar replacements within the sparse local chemical space at the required Tanimoto range (0.8–1.0). For small-molecule-dominated datasets, the method remains applicable but requires selecting a noise level suitable for the available chemical space, a consideration that should be accounted when applying this method (see SI). This small molecule limitation is shared by any molecular perturbation-based approach.

Finally, our mitigation approach is most effective against low-to-moderate resource agents, who are likely to lack the resources to de-noise, modify, or regenerate chemical datasets. High-resource actors could potentially generate novel datasets, though this requires substantial effort compared to using readily available data.

Future work should address the methodological limitations and expand the validation scope, particularly with CBRNE-relevant datasets, though very few are publicly available. First, strategies are needed to preserve utility in legitimate applications while maintaining selective censoring effectiveness, particularly for datasets with complex multidimensional risk profiles. Second, further adversarial evaluation is essential to test the robustness of feature noise and label noise, including resistance to denoising attacks, transfer learning with more sophisticated models, and detection of applied noise. For instance, label noise introduces variance that may diminish in ensemble settings through variance reduction by averaging. Third, it is essential to develop mitigation strategies for classification tasks, as many public CBRNE-relevant datasets are classification-based rather than regression tasks like those examined in this work. Finally, multi-task learning scenarios involving mixed sensitive and non-sensitive properties remain unexplored in this work but represent an important future direction.

## 7 Conclusion

The selective noise approach provides a way to censor sensitive data, helping to mitigate dual use risks in predictive chemistry (DURPC). This study investigates how adding selective noise can influence model performance, with the goal of reducing accuracy in sensitive regions while preserving it elsewhere for legitimate purposes. As discussed in the Theory section, label noise directly controls variance but does not affect bias in the sensitive region, while possibly increasing some variance in the non-sensitive region. Meanwhile, feature noise provides greater control over which regions experience accuracy reduction and higher systematic error, with its effect becoming more pronounced as the proportion of sensitive information in the dataset grows.

We have demonstrated that filtering out sensitive information alone does not prevent extrapolation in deep learning models for regression tasks, making omission ineffective for reducing model accuracy in sensitive chemical spaces. Our findings suggest that introducing feature noise during training may provide a more targeted way to influence model behavior in sensitive regions, though further research is needed to refine this approach.

While this work does not fully resolve dual use concerns in machine learning for chemistry, it demonstrates an initial step toward understanding how data perturbations affect model performance and building responsible machine learning models. We hope this work will open possibilities for future DURPC research work and contribute toward safer open-source data sharing for academic research.

## Conflicts of interest

There are no conflicts of interest to declare.

## Supplementary Material

DD-005-D5DD00512D-s001

## Data Availability

The code is available at https://github.com/ur-whitelab/chem-dual-use, including the results with multilayer perceptrons and graph convolutional networks. In addition, the repository has been archived on Zenodo at https://doi.org/10.5281/zenodo.16809359. Supplementary information (SI) is available. See DOI: https://doi.org/10.1039/d5dd00512d.
