# Supplementary material for: Censoring chemical data to mitigate dual use risk
Source: Digit Discov. 2026 Jul 6. Online ahead of print. doi: 10.1039/d5dd00512d (PMC13334314; doi:10.1039/d5dd00512d)
Supplement: DD-OLF-D5DD00512D-s001 [file DD-OLF-D5DD00512D-s001.pdf]

Supplementary Information (SI) for Digital Discovery.
This journal is © The Royal Society of Chemistry 2026

Supplemental Information for "Censoring chemical data to mitigate dual use risk"

Quintina Campbell, Jonathan Herington, and Andrew D. White

1 Perturbing SMILES For GCN Feature Noise

Table S1: Mapping between feature noise level and Tanimoto similarity scores for the GCN task

SMILES Noise Level	Tanimoto Similarity Score Range
0	1.0
0.1	0.8 -1.0
0.25	0.7 - 0.8
0.35	0.6 - 0.7
0.45	0.5 - 0.6
0.55	0.4 - 0.5
0.625	0.35 - 0.4
0.675	0.3 - 0.35
0.725	0.25 - 0.3
0.775	0.2 - 0.25
0.825	0.15 - 0.2
0.875	0.1 - 0.15
0.925	0.05-0.1
0.975	0 - 0.05

To introduce feature noise to SMILES data, the original SMILES string is replaced by a new one, with the noise level controlled by selecting a range of Tanimoto similarity scores. Candidate molecules are generated by "mutating" the original molecule and selecting the first sample that falls within the desired similarity range. We sample similar candidate molecules using the "superfast traversal, optimization, novelty, exploration, and discovery" (STONED)[1] with local chemical space generation as described by Wellawatte et al.[2]. The "noise level" is a certain range of maximum distance we allow between the true molecule and the generated molecule, called the Tanimoto similarity score. The Tanimoto similarity is computed using binary Extended-Connectivity Fingerprints (ECFP) with radius of 2[3]. Noise level equals 1 minus midpoint Tanimoto similarity (Table S1), with interval ranges selected to maximize the number of noise levels within STONED's generation capabilities. Figure S1 confirms that lower similarities are more accessible than higher similarities (e.g., 0.8-1.0) with increasing mutations, consistent with Wellawatte et al. and reflecting the nature of chemical space.

To implement this approach systematically while considering these distributions, we applied a uniform algorithm to set STONED parameters that generate candidate molecules for all noise levels. Initially, we set `max_mutations` = 1, `min_mutations` = 1, and a pool size of `num_samples` = 15. If no suitable molecule is found within the target Tanimoto similarity range, the sample size increases by 10 for each iteration until a suitable candidate is found. The parameter `max_mutations` also increases by 1 every iteration for low Tanimoto scores (< 0.3). The approach is designed to expand the chemical space by increasing the number of mutations allowed, and thereby generating more dissimilar molecules, as demonstrated in Figure S1.

If no suitable candidate is found after 10 iterations of pool generation, the original data point is removed. This occurred primarily at the lowest noise level (highest similarity range, Tanimoto score between 0.8 and 1.0), where approximately 10% of the lipophilicity dataset resulted in unresolved replacements (Table S2). This is primarily due to the limited structural diversity of small molecules, which constrains the number of valid replacements within the required similarity range. Figure S2 demonstrates one example of how small molecule structures are highly sensitive to even tiny changes. As demonstrated in Figure S1, highly similar molecules have sparser distributions compared to molecules with low Tanimoto similarity, especially for small molecules.

We further investigated the applicability of the feature noise method across all noise levels on the LD50 dataset from Zhu et al.[4], a toxicity regression dataset. The LD50 dataset contains 7,385 molecules with a median size of 23 SELF-

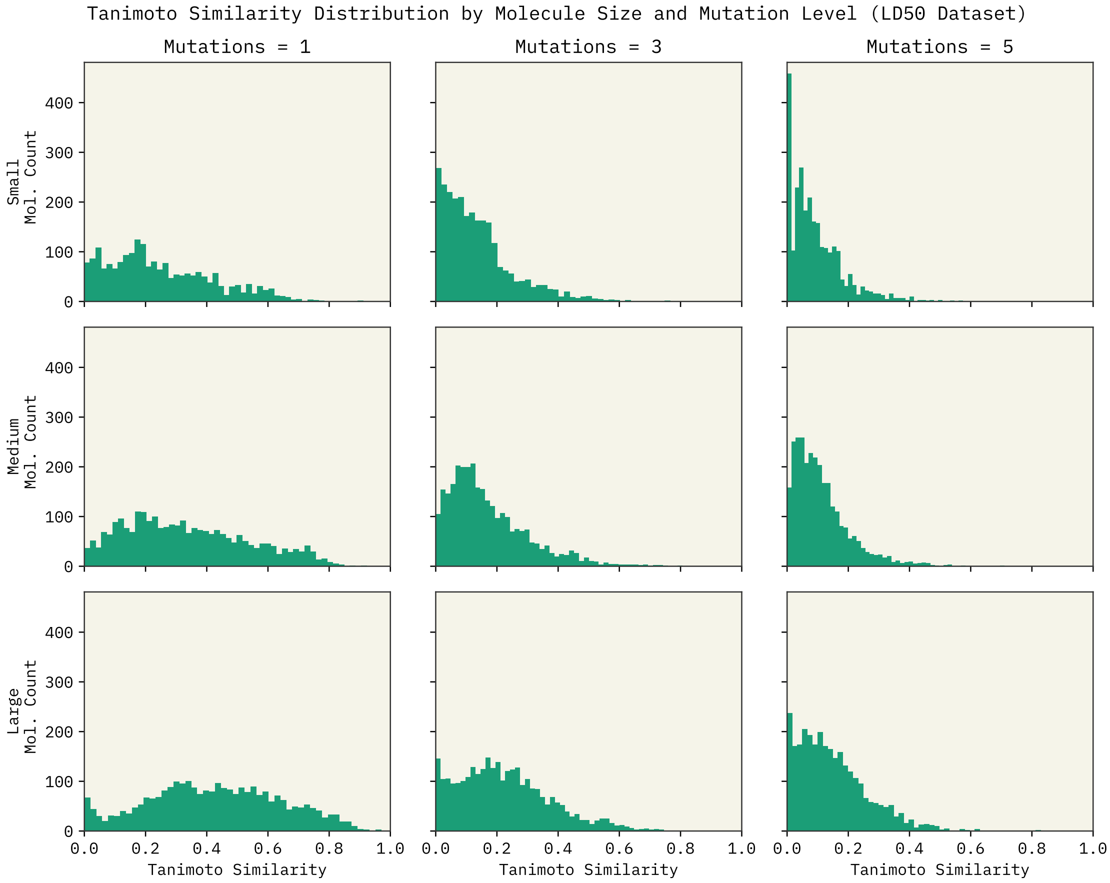

Figure S1: **Tanimoto similarity distributions by mutation count, stratified by molecule size.** To illustrate how molecule size affects the accessible chemical space in STONED, 30 molecules were randomly sampled from each size group in the LD50 dataset (Zhu et al.)[4], which spans a wide range of molecular sizes (2-167 SELFIES tokens). The resulting Tanimoto similarity distributions reflect the accessible chemical space around each molecule group. Molecules were divided into quartiles based on SELFIES token count: Small (Q1): $\leq$16 tokens; Medium (Q2-Q3): 17-31 tokens; Large (Q4): $\geq$32 tokens. STONED's internal algorithm uses SELFIES as the molecular representation.

IES tokens, considerably smaller than the lipophilicity dataset (median of 45 SELFIES tokens). Table S3 reports the number of replacement failures across Tanimoto similarity ranges when applying STONED to the full LD50 dataset. The high number of unresolved replacements is localized in a similarity range (0.8-1.0; 3,556 failures), representing nearly half of the dataset, indicating that the boundary condition has been hit at this noise level setting. The number of unresolved replacements remains substantial at mid-high range similarity ranges (0.6-0.8; 2,182 combined), consistent with the molecule-size-dependent boundary condition. In contrast, the lipophilicity dataset experiences only approximately 10% failure in this highest similarity range. The lipophilicity dataset has only 1 molecule with $<$ 10 SELFIES tokens, while the LD50 dataset has 555 molecules with $<$ 10 tokens, a substantial tail of molecules whose local chemical space is too sparse to support high-similarity perturbations. This small molecule limitation is shared by any molecular perturbation-based approach, not specific to the STONED method. Since we remove data points whose replacements are unresolved, choosing the highest similarity range (lowest noise level) for small molecules essentially degrades feature noise toward omission. For datasets with a large proportion of smaller molecules, this method remains applicable but requires selecting a noise level that accounts for the molecule size distribution. For instance, choosing a noise level corresponding to Tanimoto score 0.5-0.6 yields an unresolved replacement rate of 2.6% for the entire LD50 dataset, making the method usable as feature noise. As a molecular perturbation-based method, this STONED approach has operating conditions dependent on molecule size.

Naturally, molecular replacements have their own properties. Feature noise is induced by perturbing SMILES strings in a chemistry-aware manner while keeping labels unchanged, rather than applying neutral mathematical noise. This

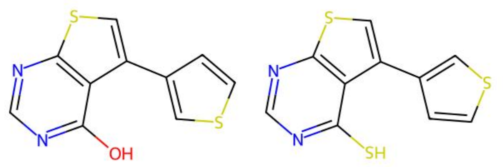

(a) (b)

Figure S2: The left is the original SMILES found in Lipophilicity dataset[5]. The right is closest similar molecule generated by STONED method[2], with Tanimoto score of 0.76.

Table S2: Number of feature noise replacement failures per Tanimoto similarity score range for the lipophilicity dataset (N=4200). The maximum number of tries was set to 10. Unresolved replacements are concentrated at the highest similarity range (0.8–1.0), consistent with the difficulty of finding highly similar replacements in chemical space.

**Score Range**	**Unresolved Replacements**	**Failure Rate (%)**
0.8-1.0	417	9.9
0.7-0.8	60	1.4
0.6-0.7	11	0.3
0.5-0.6	0	0
0.45-0.5	6	0.1
0.4-0.45	4	0.1
0.35-0.4	0	0
0.3-0.35	0	0
0.25-0.3	0	0
0.2-0.25	0	0
0.15-0.2	0	0
0.1-0.15	0	0
0.05-0.1	0	0
0-0.05	0	0

confounds the model particularly for property prediction tasks such as lipophilicity prediction. In this indirect way, it may act like label noise. To explore this, we computed Crippen logP for both the original molecules and their STONED-generated replacements and compared the resulting distributions. The Crippen logP is an octanol-water partition coefficient estimated from atom contributions using RDKit[6], serving as a proxy for the experimental logD. Figure S3 shows that the distribution shift of Crippen logP values is relatively small at low and moderate feature noise level. At the highest noise level, however, the actual lipophilicity of the replacement molecules diverges substantially from the lipophilicity of the original molecules. This implies that at high feature noise level, the attenuation bias observed in GCN results is likely accompanied by additional variance induced by both noise in features and indirect noise in labels.

Table S3: Number of feature noise replacement failures per Tanimoto similarity score range for the LD50 dataset (N=7385, Zhu et al.). The maximum number of tries was set to 10. Unresolved replacements are substantially higher across mid to high similarity ranges, reflecting the sparse local chemical space of smaller molecules.

**Score Range**	**Unresolved Replacements**	**Failure Rate (%)**
0.8-1.0	3556	48.2
0.7-0.8	1462	19.8
0.6-0.7	720	9.7
0.5-0.6	194	2.6
0.45-0.5	380	5.1
0.4-0.45	190	2.6
0.35-0.4	48	0.6
0.3-0.35	130	1.8
0.25-0.3	30	0.4
0.2-0.25	31	0.4
0.15-0.2	6	0.1
0.1-0.15	0	0
0.05-0.1	0	0
0-0.05	25	0.3

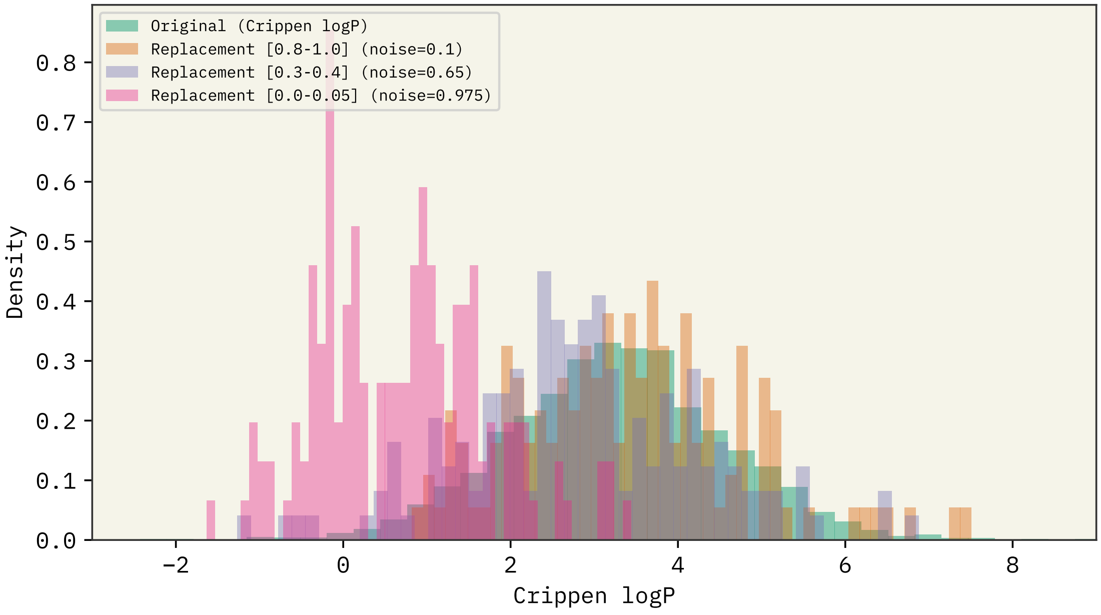

Figure S3: **Lipophilicity Distribution Shift Induced by Feature Noise** Histogram of Crippen logP of original molecules overlaid with Crippen logP of STONED-generated replacements at selected similarity ranges and corresponding feature noise levels.

2 Model & Training specifications

Table S4: Hyperparameters for Deep Learning Models

Hyperparameter	MLP	GCN
Dimension of Features (D_{in})	50	n/a
Learning Rate (lr)	0.001	0.005
Hidden Dimension	64	128
Batch Size	32	32
Data Size	6400	4200
Split (Test/Val/Train)	10/10/80%	10/10/80%
Epochs	60	200
Patience for Early Stopping	5	10
Minimum Delta (min_delta) for Early Stopping	1×10^{-4}	1×10^{-4}

The hyperparameters used during model training for multilayer perceptrons (MLP) and graph convolutional networks (GCN) are listed in Table S4. All parity plots in this paper were plotted using 'original' data, i.e. no noise addition.

To evaluate the effectiveness of selective noise in censoring sensitive data, we assess model performance on three tasks: 1) polynomial regression with 1D synthetic data, 2) multilayer perceptron (MLP) on high-dimensional synthetic data, and 3) a graph convolutional network (GCN) on an experimental molecular dataset to predict lipophilicity. For each task, we measure the performance of applying selective noise numerically and visualize the results using parity plots (y vs. $\hat{f}(x)$).

1-D Polynomial Regression We perform 1D polynomial regression on synthetic data $\mathcal{D} = \{(x_i, y_i)\}$. 200 data points were generated from the cubic equation $y = x^3 - x^2 + \epsilon$, where $\epsilon \sim \mathcal{N}(0, 0.5)$ to mimic inherent noise, evenly distributed in the domain $[-2, 3]$. For each of 100 trials, we randomly sampled 25 points to form a training set. The sensitive data were defined using a threshold $y_t = 0$, classifying the points with $y > 0$ or $y < 0$ as in the sensitive region ($s(y) = 1$). We applied three types of selective noise: feature noise ($\delta x \sim \mathcal{N}(0, 0.5)$), label noise ($\delta y \sim \mathcal{N}(0, 5)$), and combined noise ($\delta x \sim \mathcal{N}(0, 0.25)$, $\delta y \sim \mathcal{N}(0, 2.5)$). A baseline method omitted all sensitive data points and used only non-sensitive data for regression. The noisy training set was then fitted to the cubic equation using least squares.

In the baseline method, which involves omission of data points, no re-selection is conducted to compensate for these omissions. Consequently, this typically results in the analysis involving fewer than 25 data points.

Multilayer Perceptron We applied selective noise to a synthetic dataset $\mathcal{D} = \{(\vec{x}_i, y_i)\}$ and trained a multilayer perceptron (MLP) on the noisy data. Across five random seeds, we generated a dataset with 500 points, each with 50 input features, using a separate generative MLP. The sensitive region, $s(y) = 1$, was defined using three different sensitivity splits: 10%, 50%, and 90%. As described above, y_t is the label value that separates the top fraction α of the dataset, where α corresponds to the sensitivity split. Sensitive data points with $y > y_t$ in training and validation sets had Gaussian noise applied to their features and/or labels. Feature noise levels ranged from 0 to 2, and label noise levels ranged from 0 to 10. Both generative and predictive MLPs had 2 hidden layers and used a rectified linear unit (ReLU) activation function. The initial weights were randomized, differing between generative and predictive MLPs. All MLP models in all censoring methods (omission, feature noise and label noise), sensitive data proportions (10%, 50%, and 90%) and noise levels converged before the 60-epoch limit, with early stopping triggered in every trial.

Graph Convolutional Network We trained a graph convolutional network (GCN) on the lipophilicity dataset from MoleculeNet, originally curated from ChEMBL[5,7]. We labeled the dataset into sensitive and non-sensitive regions based on logP thresholds of $y_t = 3.51$, 2.33, and 0.4, for sensitivity splits at 10%, 50%, and 90%, respectively. Similarly to the MLP study, we apply selective noise to training and validation data and then measure model accuracy to predict unseen data points with no noise added. Feature noise was added by replacing SMILES within a specified Tanimoto similarity range and label noise using zero-mean Gaussian noise. The SMILES strings were then converted to molecular graphs using the canonical node featurizers from the DGL-LifeSci library, which featurizes using atom-related properties such as atom type, atom degree, hydrogen atom counts, radical electrons, formal charge, hybridization, and aromaticity[8]. The GCN model is built with DGL (Deep Graph Library)[9], consists of two graph convolutional layers with the same hidden dimensions for each. The ReLU activation function was assigned after each graph convolutional layer. After that, the readout is done by averaging the node features, and the dense layer as the final layer gives a scalar output. To isolate the effect of data perturbation despite the high inherent noise in

the dataset and model, the training, validation, and test sets are randomly split between all GCN experiments. Of all experimental conditions tested, only the omission experiments produced runs that reached full training without early stopping, with just 11 such cases identified in at most 2 trials per omission fraction: 5 in the 10% sensitive data, 1 in the 50% sensitive data, and 5 in the 90% sensitive data. All runs under both feature noise and label noise conditions triggered early stopping without exception.

Adversarial Fine-tuning of Graph Convolutional Networks We fine-tuned all layers of the GCN models on the sensitive region of the lipophilicity dataset using the same architecture and hyperparameters as the original training, with the exception of a reduced learning rate of 0.001. Models under the most challenging conditions (censor split of 0.9 at maximum noise levels or full omission) were rerun with a maximum of 500 epochs. Overall, only three runs out of all fine-tuning experiments did not trigger early stopping during fine-tuning. Fine-tuning training data was drawn exclusively from the unnoised sensitive region, while the full validation split with clean labels was retained for early stopping to ensure a stable validation loss at low clean data fractions.

3 Effect of Censoring Level on Adversarial Recovery

For the adversarial aspect of our study, we tested selected noise levels and omission fractions across all fractions of clean sensitive data available for targeted fine-tuning. Full results are shown in Figure S4. The zero clean fraction case is also included, where the noised model is evaluated without any fine-tuning, serving as the pre-recovery baseline. In the main paper, we report results at maximum censoring intensity per strategy (label noise = 5.0, feature noise level = 0.975, omission = 100%), while this figure includes additional intermediate censoring levels to show the overall recovery landscape.

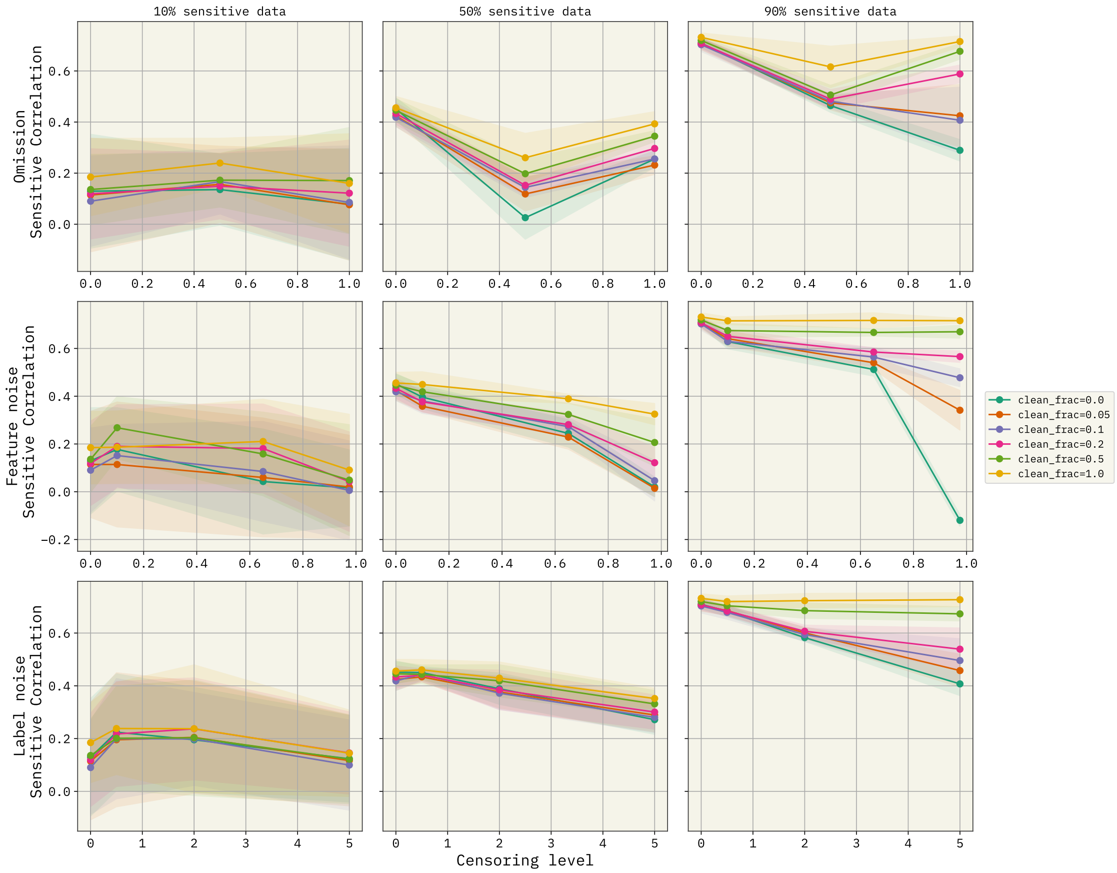

Figure S4: **Adversarial Recovery of Sensitive Region Correlation Across Censoring Levels.** Spearman correlation in the sensitive region after adversarial fine-tuning, shown as a function of censoring level (either noise level or omission fraction) for each noise type (rows) and sensitive data fraction (columns). Lines represent increasing fractions of clean sensitive data available for fine-tuning, where `clean_frac=0.0` corresponds to the noised model with no fine-tuning. Shaded bands indicate ± 1 standard deviation over 5 trials.

References

[1] AkshatKumar Nigam, Robert Pollice, Mario Krenn, Gabriel dos Passos Gomes, and Alan Aspuru-Guzik. Beyond generative models: superfast traversal, optimization, novelty, exploration and discovery (STONED) algorithm for molecules using SELFIES. *Chemical science*, 12(20):7079–7090, 2021.

[2] Geemi P Wellawatte, Aditi Seshadri, and Andrew D White. Model agnostic generation of counterfactual explanations for molecules. *Chemical Science*, 13(13):3697–3705, 2022.

[3] David Rogers and Mathew Hahn. Extended-connectivity fingerprints. *Journal of Chemical Information and Modeling*, 50(5):742–754, 2010. doi: 10.1021/ci100050t. URL `https://pubs.acs.org/doi/10.1021/ci100050t`.

[4] Hao Zhu, Todd M. Martin, Lin Ye, Alexander Sedykh, Douglas M. Young, and Alexander Tropsha. Quantitative structure- activity relationship modeling of rat acute toxicity by oral exposure. *Chemical research in toxicology*, 22(12):1913–1921, 2009.

[5] Zhenqin Wu, Bharath Ramsundar, Evan N Feinberg, Joseph Gomes, Caleb Geniesse, Aneesh S Pappu, Karl Leswing, and Vijay Pande. MoleculeNet: a benchmark for molecular machine learning. *Chemical Science*, 9(2): 513–530, 2018.

[6] Greg Landrum et al. RDKit: Open-source cheminformatics. URL `http://www.rdkit.org`.

[7] Thomas N. Kipf and Max Welling. Semi-supervised classification with graph convolutional networks, 2017.

[8] Mufei Li, Jinjing Zhou, Jiajing Hu, Wenxuan Fan, Yangkang Zhang, Yaxin Gu, and George Karypis. DGL-LifeSci: An open-source toolkit for deep learning on graphs in life science. *ACS Omega*, 6(41):27233–27238, 2021.

[9] Minjie Wang, Da Zheng, Zihao Ye, Quan Gan, Mufei Li, Xiang Song, Jinjing Zhou, Chao Ma, Lingfan Yu, Yu Gai, Tianjun Xiao, Tong He, George Karypis, Jinyang Li, and Zheng Zhang. Deep Graph Library: A graph-centric, highly-performant package for graph neural networks, 2020.
